# New Born Calf Serum Can Induce Spheroid Formation in Breast Cancer KAIMRC1 Cell Line

**DOI:** 10.3389/fmolb.2021.769030

**Published:** 2021-12-24

**Authors:** Rizwan Ali, Sarah Huwaizi, Alshaimaa Alhallaj, Arwa Al Subait, Tlili Barhoumi, Hajar Al Zahrani, Abdullah Al Anazi, Abdul Latif Khan, Mohamed Boudjelal

**Affiliations:** ^1^ Medical Research Core Facility and Platforms, King Abdullah International Medical Research Center (KAIMRC), King Saud bin Abdulaziz University for Health Sciences (KSAU-HS), MNGHA, Riyadh, Saudi Arabia; ^2^ Department of Pathology and Laboratory Medicine, King Abdulaziz Medical City (KAMC), MNGHA, Riyadh, Saudi Arabia

**Keywords:** breast cancer, 3D cell culture methods, spheroid, new born calf serum, KAIMRC1

## Abstract

Three-dimensional (3D) cell culture systems have become very popular in the field of drug screening and discovery. There is an immense demand for highly efficient and easy methods to produce 3D spheroids in any cell format. We have developed a novel and easy method to produce spheroids from the newly isolated KAIMRC1 cell line *in vitro*. It can be used as a 3D model to study proliferation, differentiation, cell death, and drug response of cancer cells. Our procedure requires growth media supplemented with 10% new born calf serum (NBCS) and regular cell culture plates to generate KAIMRC1 spheroids without the need for any specialized 3D cell culture system. This procedure generates multiple spheroids within a 12–24-h culture. KAIMRC1 spheroids are compact, homogeneous in size and morphology with a mean size of 55.8 µm (±3.5). High content imaging (HCI) of KAIMRC1 spheroids treated with a panel of 240 compounds resulted in the identification of several highly specific compounds towards spheroids. Immunophenotyping of KAIMRC1 spheroids revealed phosphorylation of FAK, cJUN, and E-cadherin, which suggests the involvement of JNK/JUN pathway in the KAIMRC1 spheroids formation. Gene expression analysis showed upregulation of cell junction genes, GJB3, DSC1, CLDN5, CLDN8, and PLAU. Furthermore, co-culture of KAIMRC1 cells with primary cancer-associated-fibroblasts (CAFs) showcased the potential of these cells in drug discovery application.

## Introduction

Globally, breast cancer is one of the leading causes of death in women. Many risk factors have been identified, but the real cause of this malignancy has not been discovered yet. There are many types of breast cancer, and these types differ in their ability to metastasize to other parts of the body. In basic research, cell lines that are riddled with genetic mutations are the most valuable tool for researchers to employ to understand the mechanism of initiation and spreading of any type of cancer, including breast cancer. There are several breast cancer cell lines available globally to study this malignancy. However, cell lines are traditionally grown in two dimensions (2D) and do not precisely mimic the real physiology of the breast tissue. 2D monolayered cell culture systems represent only a simplified version of the cancer environment. Researchers have been trying for decades to establish an *in vitro* system that can mimic the actual *in vivo* like breast tissue environment.

Over the past few years, three-dimensional (3D) cell culture systems have earned tremendous attention, as they combine the advantages and ease of *in vitro* work with the microanatomy found *in vivo*. These 3D cell cultures are often termed organoids, tumoroids, or spheroids, and they closely mimic the tissue or organ from which they are derived. Spheroids are grown as floating spheres and differ from each other in the source of cells, method, and cultivation time. They are considered a valuable tool in early drug discovery and have already been proposed for potential therapeutics (Bauman et al., 2018). For instance, spheroids were initially developed in the 1970s by Sutherland and coworkers (Sutherland et al., 1970; Sutherland et al., 1971), and there onwards, spheroids have been increasingly employed in experimental cancer research as mono- or co-cultures with different types of cells. The interest in growing and utilizing spheroids is increasing rapidly, as researchers realize that the data generated using spheroid culture are closer to reality than by 2D cultures. Soon, spheroids consisting of multiple cell types grown on a specialized matrix mimicking the real 3D environment will be available to the researchers to better understand the onset of many diseases, including breast cancer.

Lately, there has been a noteworthy increase in scientific literature bringing up new spheroid culture methods. These methods are used to generate mammospheres (Lombardo et al., 2015), hepatospheres (Lucendo-Villarin et al., 2019), and neurospheres (Soares et al., 2020). These spheroids are cheaper, relatively easier, and faster to produce. For example, a recent report described spontaneous spheroid budding from monolayers of ovarian cells and correlated it with the lack of cortical E-cadherin and the abundance of vimentin (Pease et al., 2012). Another research presented the tripeptide, RGD (arginine–glycine–aspartic acid) induced self-assembly to generate multicellular spheroids by directly adding RGD to cell monolayers (Akasov et al., 2016). It is known that cancer cells degrade the extracellular matrix (ECM) to metastasize. The tumor-derived basement membrane extract (BME/Matrigel) has also been utilized to study ECM-based aggregation and invasiveness of the cells (Benton et al., 2011). The malignant potential of the cells can also be predicted using this technique (Albini et al., 1987).

It is well established that cancer cell lines have a subpopulation of cancer stem cells (CSCs) (Al-Hajj et al., 2003). The number of CSCs differs from one cell line to another. These CSCs are instrumental in the progression of cancer and metastasis (Shiozawa, 2013; Reynolds et al., 2017; Ayob and Ramasamy, 2018). Tumor-derived spheroids are thought to be enriched in CSCs (Ishiguro et al., 2017), and it has been demonstrated that even a single CSC can initiate their formation (Sato et al., 2009). The key to growing spheroids lies in the use of a nutrient-rich medium. This medium enhances the growth of stem cells within the spheroids, just like *in vivo*, where stem cells grow and proliferate in specialized niches.

Recent publications show a developing trend of spheroids biobanking by growing spheroids from different organs of the body, for example, liver, breast, pancreas, stomach, brain, and the prostate (Van De Wetering et al., 2015; De Angelis et al., 2018). These spheroids can later be used in developing personalized medicines (Hagemann et al., 2017; Colella et al., 2018) by screening against a panel of drugs to determine their efficacy on specific organs. In this regard, high throughput screening (HTS) has been utilized efficiently. HTS is a critical step in lead identification using compound libraries. Most of the cell-based assays are designed on 2D cell culture systems that are not mimicking the actual 3D physiological environment. In this context, 3D cell culture systems are gaining popularity to replace 2D systems. The identified drugs are thought to be more reliable and can then be used in animal models and clinical trials. Nonetheless, in the future, the development of reliable *in vitro* 3D assays and safe, transplantable, and preclinical validated 3D cell models will be instrumental in developing precise therapeutics.

Not so long ago, we established a naturally transformed breast cancer cell line, KAIMRC1, from an Arab woman suffering from ductal breast carcinoma (Ali et al., 2017). These cells have been characterized for the biological and molecular markers, induction of MAPK pathways, nuclear receptors (Nehdi et al., 2019), exome, (Rashid et al., 2021) proteome (Alghanem et al., 2020), and its response to different commercially available drugs and compounds. Furthermore, KAIMRC1 cells have a remarkable ability to make 3D spheroids in cell culture media without the help of specialized carriers and expensive growth-enhancing reagents. In this work, we have characterized these spheroids to study their growth behavior, drug response, and gene and protein pathways.

## Materials and Methods

### Cell Lines Culture

Human breast cancer epithelial cell lines, MDA MB-231 (HTB26), and MCF-7 (HTB-22), and human non-tumorigenic breast epithelial cell line, MCF-10 A (RL-10317), were purchased from ATCC, United States. All the cell lines, including KAIMRC1, were maintained in advanced Dulbecco’s modified Eagle medium (DMEM) (Invitrogen) supplemented with 10% fetal bovine serum (FBS) (FBS-DMEM), 50 U/ml penicillin (Gibco) and 50 μg/ml streptomycin (Gibco), and 2 mM L-glutamine (Gibco) at 37°C in a humidified 5% CO_2_ atmosphere. MCF-10A was maintained in 1:1 DMEM/F12 media (Invitrogen) supplemented with 5% equine serum, 0.5 μg/ml hydrocortisone, 100 ng/ml cholera toxin, 10 μg/ml insulin, 20 ng/ml epidermal growth factor (EGF), 50 U/ml penicillin and 50 μg/ml streptomycin, and 2 mM L-glutamine. Cells were cultured at 37°C in a humidified 5% CO_2_ atmosphere.

### Spheroid Culture

KAIMRC1 cells were cultured in a 96-well cell culture plate (Nunc, Thermo Fisher Scientific) at a density of 10,000 cells/well. One half of the plate was incubated with advanced DMEM supplemented with FBS to generate KAIMRC1 monolayer, and the other half was incubated with advanced DMEM supplemented with 10% new born calf serum (NBCS), (NBCS-DMEM), to generate KAIMRC1 spheroids, at 37°C in a humidified 5% CO_2_ atmosphere. The following day, cell culture plate was monitored under a microscope to look for spheroids formation before any downstream experiments.

### Cancer-Associated Fibroblasts Isolation

Human breast cancer tumor samples were collected after obtaining the informed written patients’ consent following the guidelines set by the KAIMRC Ethics Committee. The sample was washed several times with phosphate-buffered saline (PBS), minced and cut into smaller pieces, and incubated normal cell culture medium in 24-well cell culture plates at 37°C in a humidified environment. The tissue culture was monitored microscopically on a daily basis to look for adherent fibroblast-like cells protruding out of the tissue pieces. After several days of culture, tissue pieces were removed, and the adherent cells were trypsinized and subcultured. These cells were incubated with fibroblast monoclonal antibody (D7-FIB, MA5-16642, Invitrogen) and α-smooth muscle actin (1 μg/ml, Abcam, UK), overnight at 4°C. It was followed by Alexa Fluor 488-conjugated secondary antibody at room temperature for 1 h. The cells were analyzed using flow cytometry. These cells were further passaged and used in the co-culture studies.

### Co-Culture of CAFs and KAIMRC1 Cells

Primary breast CAFs were isolated as described above and cultured in a 96-well plate at 5,000 cells/well. At 90% confluency, cells were stained with Live CellTracker™ Orange CMTMR dye (10 µM, Cat. No. C2927, Invitrogen) for 1.5 h in a humidified incubator. At the same time, KAIMRC1 cells were subcultured and stained with Live CellTracker™ Green CMFDA dye (2.5 µM, Cat. No. C7025, Invitrogen) for 45 min in a humidified incubator. Both CAFs and KAIMRC1 were washed 2x with PBS and stained with HOECHST 33342, trihydrocholoride, trihydrate (1 μg/ml, Cat. No. H3570, Invitrogen) for 15 and 5 min, respectively. KAIMRC1 cells were then seeded on the CAFs monolayer at a density of 2,500 cells/well. This co-culture was then monitored every day for 6 days, and images were acquired using the Zeiss LSM780 microscope system.

### Size Distribution Assay

Single KAIMRC1 cell spheroids were cultured as described above in a six-well plate, and images were acquired every 24 h for 14 days to measure the size distribution of the spheroids under an EVOS FL auto system equipped with live-cell imaging capabilities. We counted at least 40 spheroids per well to demonstrate the size distribution of KAIMRC1 spheroids.

### Compound Treatment and Cell Viability Assay

To study the effect of compound treatment, KAIMRC1 monolayer and spheroids were treated with a large panel of commercially available compounds. Briefly, KAIMRC1 cell monolayer and spheroids were seeded at 5,000 cells/well into 96-well plates and incubated at a 5% CO_2_ incubator overnight. Subsequently, cells were treated with compounds in serial dilution followed by incubation for 48 h. 3-(4,5-Dimethylthiazol-2-yl)-2,5-diphenyltetrazolium bromide (MTT) assay was performed after 48 h. Five microliters of MTT (5 mg/ml) was added to the cell culture wells and incubated for 4 h. The plate was centrifuged at 500x*g* for 5 min to pull down all the floating spheroids. Later, the media was aspirated carefully avoiding the disruption of spheroids. The plates were then dried on paper towels followed by the addition of dimethyl sulfoxide (DMSO). The plates were incubated with DMSO on a shaker for 1.5 h; the spheroid wells were observed under a microscope to look for any unsolubilized spheroids. The absorbance was read at 560 nm using a Molecular Devices Spectra Max Plus 384 spectrophotometer. Background readings were collected at 620 nm. Absorbance readings were normalized and expressed as a relative percentage. The data were analyzed in GraphPad Prism 8 software, and the half-maximal inhibitory concentration (IC_50_) was determined. Error bars denote standard deviation (SD). The statistical significance of differences was determined using the Students’ t-test. *p* < 0.05 was considered statistically significant.

### High Content Imaging Assay

KAIMRC1 spheroids and monolayers were plated in 96-well plates at a density of 5,000 cells per well. Cells were treated with graded concentrations of Mitoxantrone ranging from 50 to 0 µM for 48 h. Post-treatment, cells were stained with calcein AM (2 μg/ml), HOECHST33342 (2.5 μg/ml), and propidium iodide (2.5 μg/ml) for 10 min at 37°C and 5% CO_2_. Plates were imaged using a Molecular Devices ImageXpress^®^ Micro and analyzed using MetaXpress^®^ software, Molecular Devices, Downingtown, PA, United States. Live/Dead module available in the MetaXpress software was used to count live and dead cells. The acquired values were fed in the GraphPad Prism 8 software to calculate IC_50_. All experiments were performed in triplicates.

### RNA Isolation and cDNA Reverse Transcription

Total RNA isolation from KAIMRC1 cells and spheroids was performed using an RNA Isolation Kit (Ambion) following manufacturer instructions, and RNA was quantified with a Nanodrop 8000 spectrophotometer (Thermo Scientific). One microgram of total RNA was reverse transcribed into cDNA using High Capacity cDNA Reverse Transcription Kit (Applied Biosystems) and was diluted 1:2 in *dd*H_2_O.

### Real-Time qPCR of Qiagen Breast Cancer and Cell Junction Panel

The manufacturer’s protocol was followed for both the panels. The PCR amplification was performed on a 7900HT Fast Real-Time PCR System (Applied Biosystems). The data was analyzed in RQ Manager 1.2.1 software (Applied Biosystems), and relative changes in mRNA expression levels were calculated by the ΔΔ_Ct_ method in the RT2 Profiler PCR Array Data Analysis Spreadsheet Version 5.1, 4/2019. It is available on Qiagen web portal.

### Immunocytochemistry and Confocal Laser Scanning Microscopy

Cells were seeded 24 h before the experiment in *µ*-slide eight-well Ibitreat chamber slides (Cat. No. 80,826, Ibidi, Germany). Cells were fixed, permeabilized, and incubated overnight at 4°C with the diluted primary mouse monoclonal antibody to β-3-tubulin (TU-20; Cat. No. 4466s), mouse monoclonal antibody to *a*-tubulin (DM1A; Cat # 3873s), rabbit monoclonal antibody to caveolin-1 (D46G3; Cat. No. 3267s), rabbit monoclonal antibody to E-cadherin (24E10; Cat. No. 3195s), rabbit monoclonal antibody to Phopho-Akt (Ser473; D9E XP^®^; Cat. No. 4060s), and β-actin (BA3R; Cat. No. MA5-15739, Thermo Fisher Scientific; 1:500). All antibodies were purchased from Cell Signaling. Secondary antirabbit Alexa Fluor^®^ 488 (H + L; Ref. No. A11034) and antimouse Alexa Fluor™ 647 (H + L; Ref. No. A21236) from Life Technologies were used to detect antibody fluorescence. Slides were imaged using cLSM 780 (Carl Zeiss, Germany). Cell Tracker™ Green (Life Technologies) and Alexa Fluor 488 were detected using argon laser at 488 nm/520–530 (ex/em), whereas Alexa Fluor 647 was detected using HeNe laser at 633 nm/650 (ex/em). The counterstained nucleus was stained with HOECHST 33342 and detected using a UV laser at 350 nm/460 nm (ex/em).

### Flow Cytometry Analysis of Cell Markers

Flow cytometry was performed using the FACS Canto II cytometer (BD), and subsequent analysis was performed using the FACS Diva software (BD). Briefly, cells and spheroids resuspended in PBS were pelleted. Fluorescently conjugated primary antibodies α-smooth muscle 1A4 (Abcam), fibroblast monoclonal antibody (D7-FIB, MA5-16642, Invitrogen), CD24, CD234 (E-Cadherin), CD44, CD227, EpCAM (EBA-1), CD49c, CD116, SSEA-1, CD45, CD47, ALDH1A1 (LSBio), cytokeratin 18 (CK18), and SSEA-1 (CD15) were purchased from BD Pharmingen unless otherwise specified. Cells were incubated with antibodies for an hour and then analyzed on the flow cytometer. For the flow cytometry analysis, cells were gated based on FSC and SSC properties, and interval gates on histogram plots were set up for positive cell populations in FITC, PE, and APC-Cy7 channels.

### Scanning Electron Microscopy

Spheroids were grown and fixed as described above. Cells were then dehydrated with graded concentrations of ethanol (Sigma) and transferred to appropriate carbon taped stubs (Ted Pella, USA) for SEM. To enhance the electron conductivity, samples were coated with gold/palladium (Au/Pd) by sputter coating and examined on an FEI NanoSEM 450 scanning electron microscope at 10 kV.

### Transmission Electron Microscopy

Monolayer and spheroids cell culture was performed as described above. The specimen was fixed in 4% glutaraldehyde in 0.1 M PBS (pH 7.4) for 1 h. After washing in the same buffer, the sample was post-fixed in 1% OsO_4_ in 0.1 M PBS (pH 7.4) for 1 h, followed by washing with PBS (3x). The specimen was then dehydrated in a series of acetone solutions and infiltrated in acetone/resin (1:1) for 1 h and then with acetone/resin (1:2) for 3 h. The specimen was then embedded in epoxy resin (Araldite) and placed in an oven at 80°C overnight to polymerize. Ultrathin sections were obtained with ultramicrotome (RMS), mounted on copper grids, and stained for contrast with heavy metal stains (uranyl acetate and lead citrate). TEM images were then collected on a JEOL-JEM 1400 operating at 120 kV using a Gatan camera with Digital Micrograph Imaging software.

### Histopathology

KAIMRC1 monolayer and spheroids samples were fixed in 4% formaldehyde, placed in cassettes, and mounted into an automated vacuum tissue processor (Leica Biosystems, Germany). The samples were embedded and blocked in paraffin wax, and thin sections were made using a microtome (Accu-Cut^®^ SRM™, Sakura, Netherlands). Sections were stained with Mayer’s hematoxylin solution and counterstained in eosin–phloxine solution. Slides were examined and imaged at 10x and 40x objectives under an upright microscope (Eclipse TS100, Nikon, Japan).

### Western Blotting

KAIMRC1 monolayer and spheroids cultures were lysed, and total protein was extracted. The Western blotting analysis was performed with primary antibodies against cell–cell and cell–matrix contact proteins, namely, rabbit monoclonal antibody to integrin β4 (Cat. No. 4707s), E-cadherin (24E10; Cat. No. 3195s), FAK (D2R2E; Cat. No. 13009s), caveolin-1 (D46G3; Cat. No. 3267s), and survivin (71G4B7; Cat. No. 2808). All the antibodies were purchased from Cell Signaling and used at 1:1,000 dilution. Signals were detected using a ChemiDoc MP System (Bio-Rad) and analyzed on ImageLab software. Sample loading was examined by probing the same membrane with an anti-GAPDH antibody.

### Human Proteome Profiler Arrays

To further profile the proteins and their phosphorylation status involved in spheroids formation, several Human Proteome Profilers were utilized. Human Phospho-Kinase Array (Catalog No. ARY003B), Human Pluripotent Stem Cell Array (Catalog No. ARY010), Human Phospho-RTK Array (Catalog No. ARY001B), and Human Phospho-Immunoreceptor Array (Catalog No. ARY004B). All were purchased from R&D Systems, Minneapolis, MN, USA, and used according to the manufacturer’s protocol.

## Results

### Growth Characteristics of Spheroids

**FIGURE 1:**
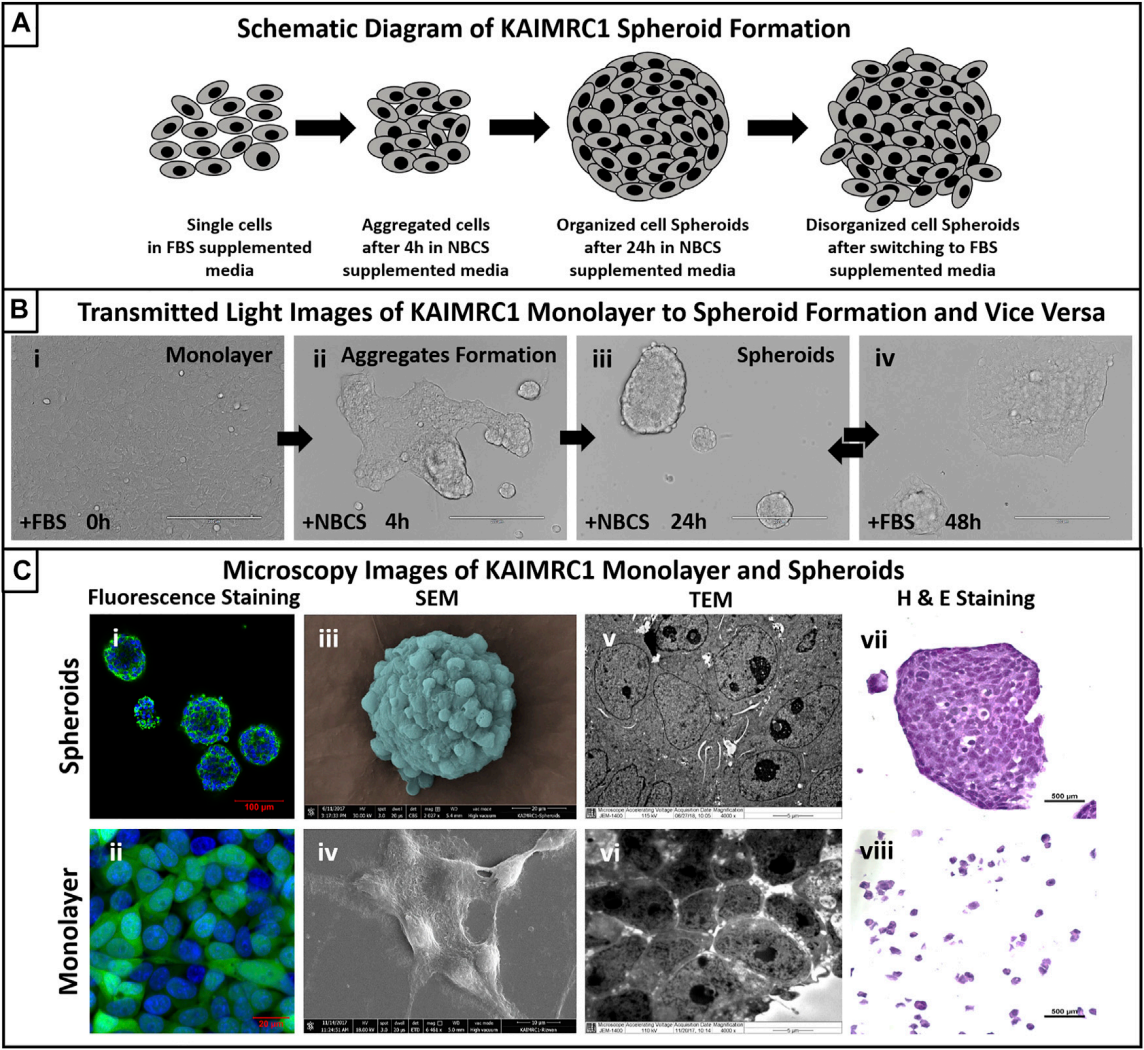
**(A)** Schematic diagram of KAIMRC1 spheroids formation. KAIMRC1 cells form loosely connected aggregates within 4 h of culture in NBCS media and organize in tightly connected spheroid in the next 24 h. These spheroids start disintegrating and form a monolayer when switched back to FBS media. **(B)** Transmitted light images of KAIMRC1 monolayer to spheroid and vice versa. (i) Monolayer of KAIMRC1 at time 0 and with normal 10% FBS DMEM, (ii) aggregate formation within 4 h of media change to 10% NBCS, (iii) spheroids formation in next 24 h in%NBCS media, (iv) formation of monolayer within 24 h of media change back to 10% FBS. (i–iv) Scale bar 200 μm. **(C)** Light and electron microscopy of KAIMRC1 cells in monolayer and spheroids. (i, ii) Post-fixation monolayer and spheroids were stained for nucleus (blue) and cytoplasm (green). Scanning electron microscope images of (iii) KAIMRC1 spheroid. Scale bar 20 μm. (iv) KAIMRC1 monolayer. Scale bar 10 μm. Transmission electron microscope image of (v) KAIMRC1 spheroid (Zoomed in) and (vi) KAIMRC1 monolayer. Tight connections between cells in spheroids is visible in comparison to monolayer. (vii, viii) Post-fixation, cells and spheroids were stained for hematoxylin and eosin Y to identify any structural related changes in KAIMRC1 cells. Cancer disrupts the normal control mechanisms of cell proliferation and differentiation that result in changes in the structure and appearance of the cells. H and E staining is critical in identifying these changes.

The growth kinetics of KAIMRC1 spheroids at passages 30–60 was studied. KAIMRC1 cells were seeded in six-well plates in FBS-DMEM until confluence was achieved. Then, the media was changed to NBCS-DMEM. Initially, within 4 h of incubation, the cell monolayer started to retract and formed tube-like structures, followed by the formation of big organized spheroids in the next 24 h. To confirm that the spheroid formation is a reversible process, we again switched to the FBS-DMEM growth media. Interestingly, spheroids started to adhere and spread once again on the plate surface within a few hours. Finally, spheroids were completely dispersed back as a monolayer in 48 h of culture (Figure 1B).

To identify the optimal percentage of NBCS required to initiate spheroid formation, we incubated KAIMRC1 cells with a graded concentration of NBCS-DMEM, i.e., 1, 2.5, 5, 10, and 20% NBCS. The first two concentrations, 1% and 2.5%, showed monolayer and aggregation of KAIMRC1 cells, but no detachment from the plate surface; 5% showed many detached spheroids along with attached aggregates; whereas 10% and 20% concentrations showed complete detachment and floating spheroids of KAIMRC1 cells (data not shown).

**FIGURE 2:**
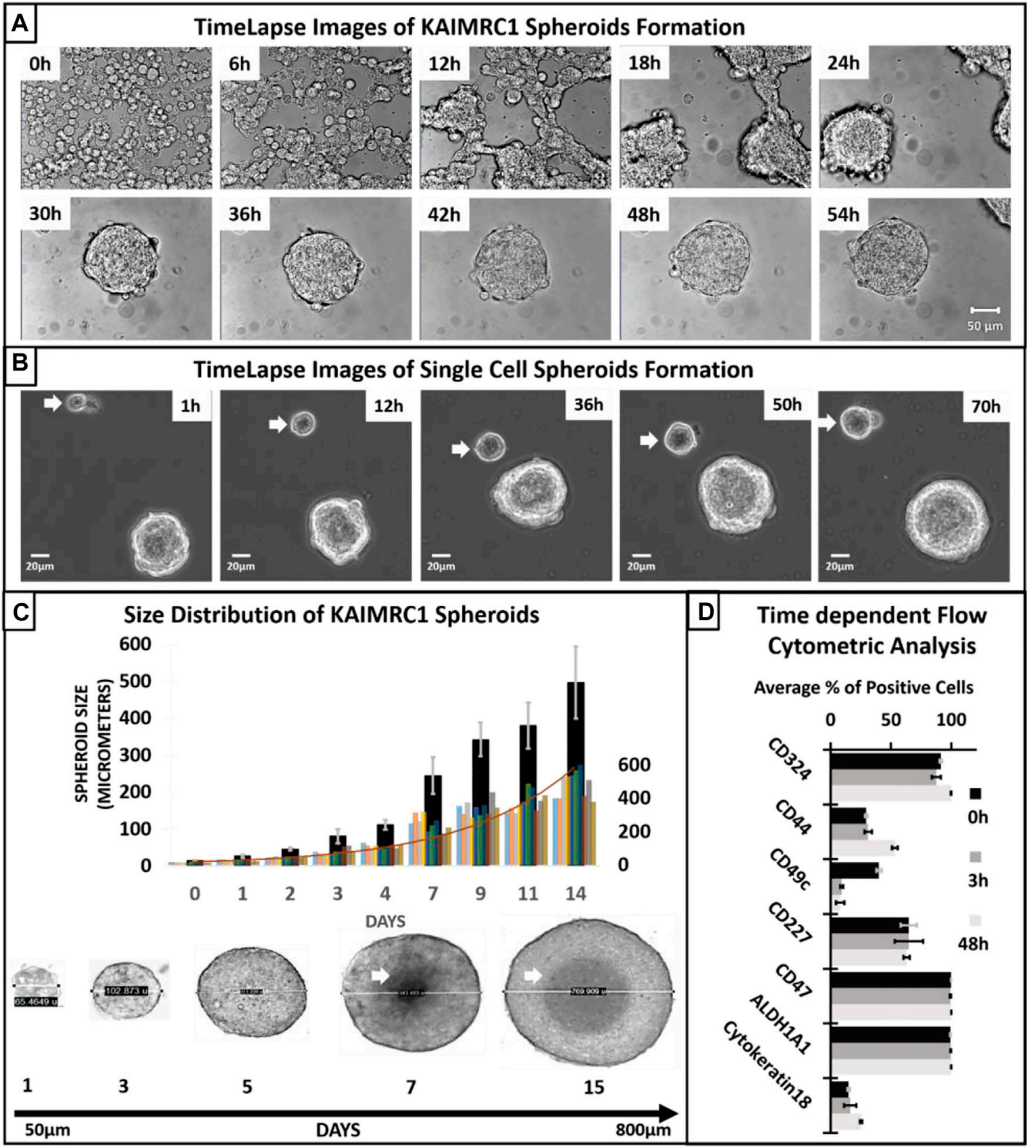
**(A)** Time-lapse transmitted light imaging of KAIMRC1 cells. Cells were cultured in DMEM containing NBCS in Ibidi 8 well µ-dish. Images were taken on Zeiss LSM 780 system at an interval of 1 h. Representative images after every 6 h are shown here. It is evident from the image at 6 h that aggregate formation started within 6 h of incubation in DMEM containing NBCS. Spheroid formation is observable within 24–30 h. All the images have a scale bar = 100 µm. **(B)** Spheroid formation from a single KAIMRC1 cell. Time-lapse imaging for 3 days shows a single cell dividing and forming a spheroid (white arrow). **(C)** Size distribution of KAIMRC1 spheroids. KAIMRC1 cells were subjected to spheroid formation for 14 days. Spheroids reached up to 800 µm. Necrotic core was visible when spheroid size crossed 300 µm i.e., around the seventh day. A histogram of all the spheroids counted for the size distribution study is overlayed. **(D)** Time-dependent flow cytometric analysis of breast cancer and stem cell-specific markers. Interestingly, there was an increase in the CD44^+^ cells at 48 h timepoint and a significant decrease in CD49c at 3 and 48 h. CD324 and Cytokeratin18 expression also increase at 48 h.

When cultured in the NBCS-DMEM condition, KAIMRC1 cells initially aggregated together. During the aggregation process, cells fuse, forming strings, and later these strings round up to form spheroids. These spheroids then increased in size for several days before reaching a critical point, i.e., around the seventh day when necrosis is visible at the center of the spheroids. At this point, spheroids are no longer organized and start disintegrating by shedding cells (Figure 2A). Most of these excluded cells are attributed to having lost essential cell–cell adhesion molecules (Stadler et al., 2018). However, few among these single cells were able to divide and make smaller spheroids (Figure 2B). Single cells are pointed out with a white arrow in Figure 2B showing spheroid formation over time.

Spheroids size distribution assay exhibited that the size of KAIMRC1 spheroids ranges from 50 to 700 µm with a mean size of 55.8 µm (SD ± 3.5), and the maximum size can be reached within 14 days of culture (Figure 2C). Notably, a necrotic center can be seen after 6 days of culture. This result suggests that an approximate size of 300 µm that can be reached in 3–4 days might be optimal to be used in drug screening applications.

**FIGURE 3:**
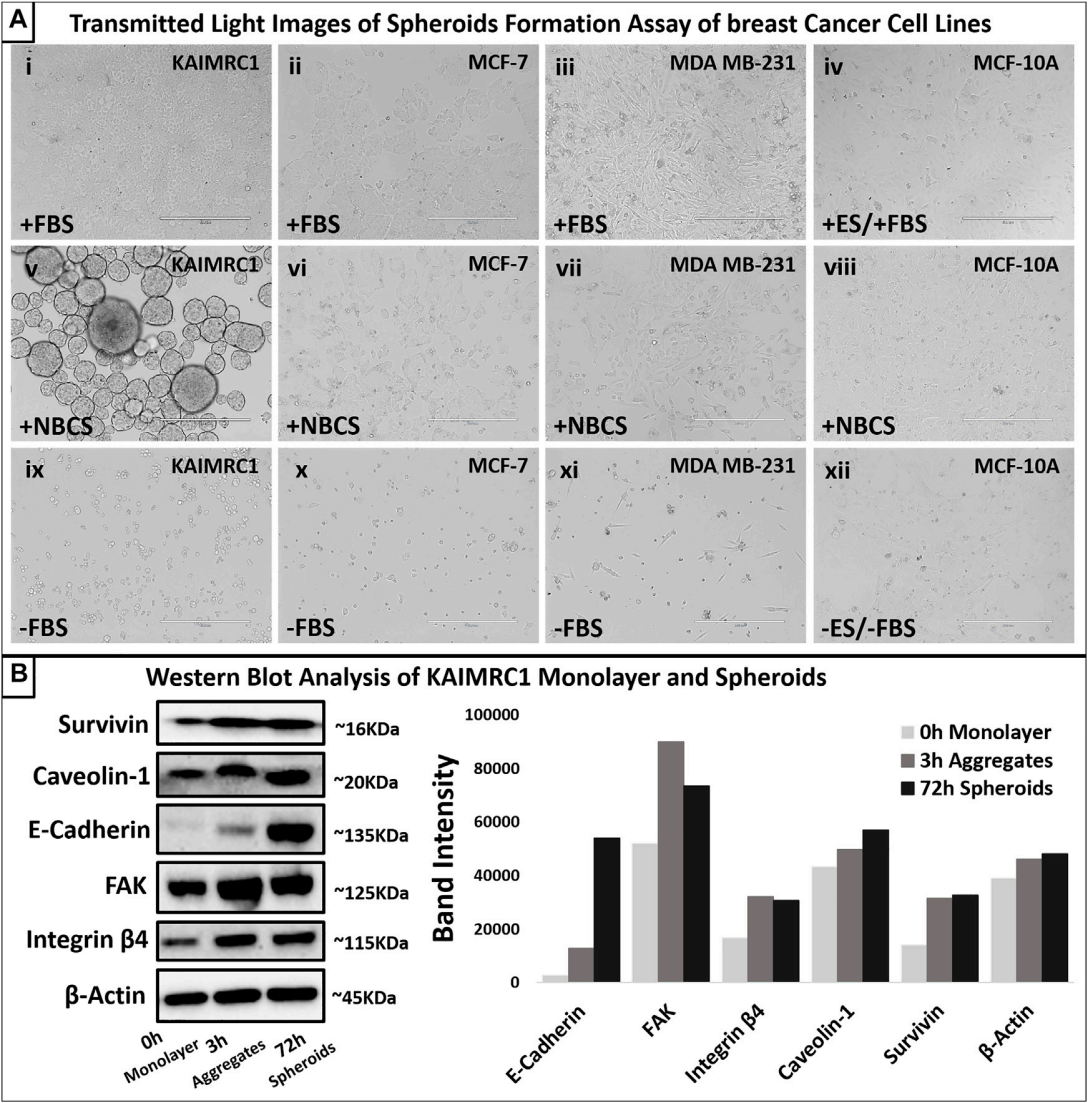
**(A)** Spheroid formation assay. Four breast cancer cell lines, KAIMRC1 MCF-7, MDA MB-231, and MCF-10A, were tested in a 12-well plate. All the cells were cultured for 4 days in DMEM. KAIMRC1, MCF-7, and MDA MB-231 cells were cultured in DMEM containing (i, ii, and iii) 10% FBS, (v, vi, and vii) 10% NBCS, and (ix, x, and xi) 0% FBS. MCF-10 A cells were incubated in DMEM containing (iv) 5% ES and 5% FBS, (viii) 10% NBCS, and (xii) 0% ES and 0% FBS. Cells cultured in normal growth media with 10% FBS showed linear proliferation over 4 days, whereas cells cultured in NBCS showed prolonged growth with cell death in all other cells except KAIMRC1. KAIMRC1 cells started making spheres from day 1 and grew in size over time. Cell death was evident in the cell cultures lacking any serum. Scale bar 400 µm. **(B)** Western blot analysis of expression of focal adhesion proteins in KAIMRC1 monolayer and spheroids. E-cadherin, FAKs, caveolin-1, survivin, and integrin β4 showed increased expression in spheroids compared to monolayer. β-Actin served as a control.

To study the effect of NBCS-DMEM on other well-established breast cancer cell lines, MDA MB-231, MCF-7, and MCF-10A, we incubated these cell lines both with FBS-DMEM and NBCS-DMEM for 48 h. Only KAIMRC1 cells showed spheroid formation in NBCS-DMEM, whereas other cell lines did not show any signs of spheroid formation (Figure 3A). Therefore, it was confirmed that NBCS is triggering spheroid formation in KAIMRC1 cells only.

### Immunophenotyping

**FIGURE 4:**
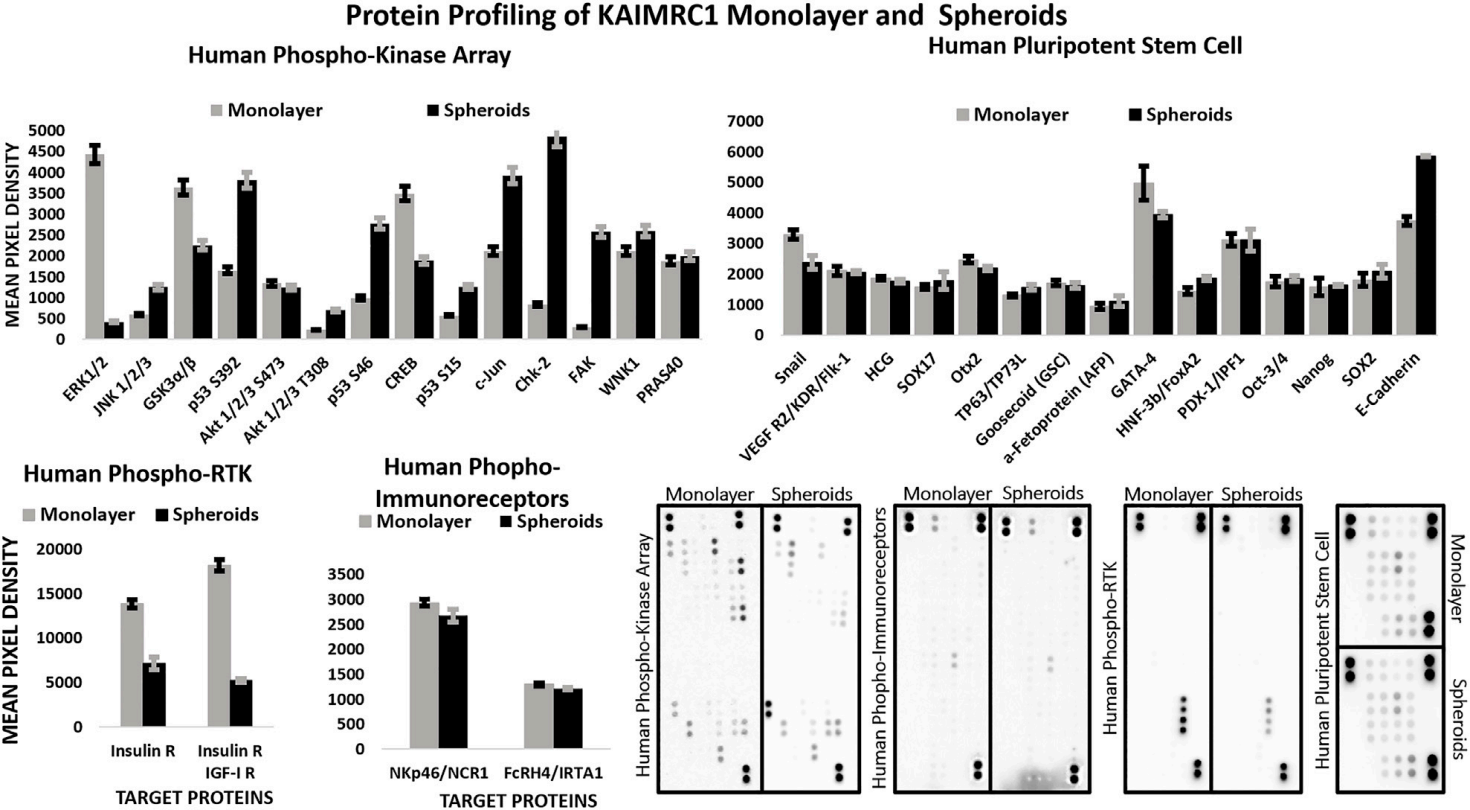
Multiplex Protein profile of KAIMRC1 monolayer and spheroids. Human phosphokinase, phosphor RTK, phospho immunoreceptors, and stem cells arrays were utilized to detect the expression and phosphorylation status of key proteins involved in breast cancer. In comparison to monolayer, spheroids showed a notable decrease in the activation of ERK1/2, GSK3 α/β, CREB, insulin receptor, and IGF-I receptor, whereas they showed an increase in the activation of p53, c-Jun, ChK-2, FAK, and E-cadherin.

**FIGURE 5:**
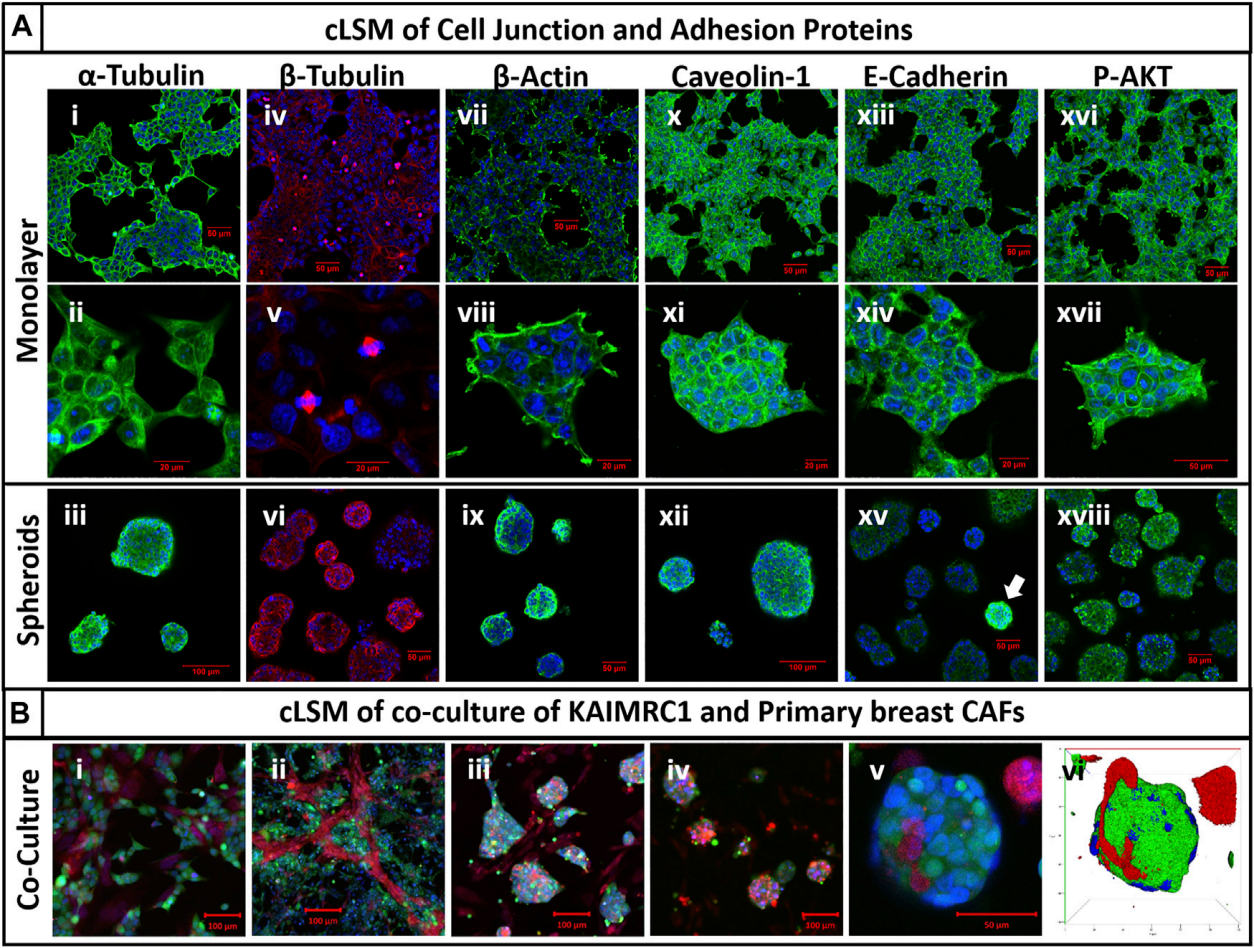
**(A)** Immunocytochemistry of cell junction and adhesion proteins of KAIMRC1 monolayer and spheroids. Cells were stained for (i–iii) α-tubulin, (iv–vi) β-tubulin, (vii–ix) β-actin, (x–xii) caveolin-1, (xiii–xv) E-cadherin, and (xvi–xviii) P-AKT. Blue = nucleus, green/red = secondary fluorescent A.B. primary protein of interest. Except for P-AKT, all other proteins showed increased expression in spheroids, which is evident from the images. White arrow shows overexpression of E-cadherin in spheroids in the image (xv). **(B)** cLSM of KAIMRC1 cells co-cultured with primary breast CAFs. Images (i–v) showcase the formation of co-cultured spheroids of KAIMRC1 with primary breast CAFs and (vi) presents a 3D reconstruction of the spheroid in the image (v). Blue = nucleus, red = CAFs, and green = KAIMRC1.

Western blot analysis of monolayer and spheroids was performed on several key cell–cell interactions and adhesion protein. Results demonstrated an increase in the expression of E-cadherin, a member of adherens junction transmembrane protein; focal adhesion kinases (FAK), a cytoplasmic tyrosine kinase protein involved in cell survival and migration; Caveolin-1, a plasma membrane protein involved in cell adhesion; Survivin, an antiapoptotic protein highly expressed in malignant cells; and integrin β4, a heterodimeric cell surface receptor involved in cell adhesion, growth, and survival. (Figure 3B). Protein profiling and immunocytochemistry also confirmed the overexpression of E-cadherin, caveolin-1, and FAK in KAIMRC1 spheroids (Figure 4 and Figure 5A).

We also performed time-dependent flow cytometric analysis of breast cancer and stem-cell-specific markers. Cells were collected at three time points 0 h (monolayer) and 3 and 48 h. Interestingly, there was an increase in the CD44^+^ cells at 48 h timepoint and significant decrease in CD49c at 3 and 48 h. CD324 and cytokeratin 18 expression also increase at 48 h.

\In comparison to the monolayer, protein profiling of spheroids also showed notable decrease in the expression of ERK1/2, GSK3 α/β, CREB, insulin receptor, and IGF-I receptor, whereas an increase in the expression of p53, c-Jun, ChK-2 (Figure 4).

### Gene Expression Analysis

**FIGURE 6:**
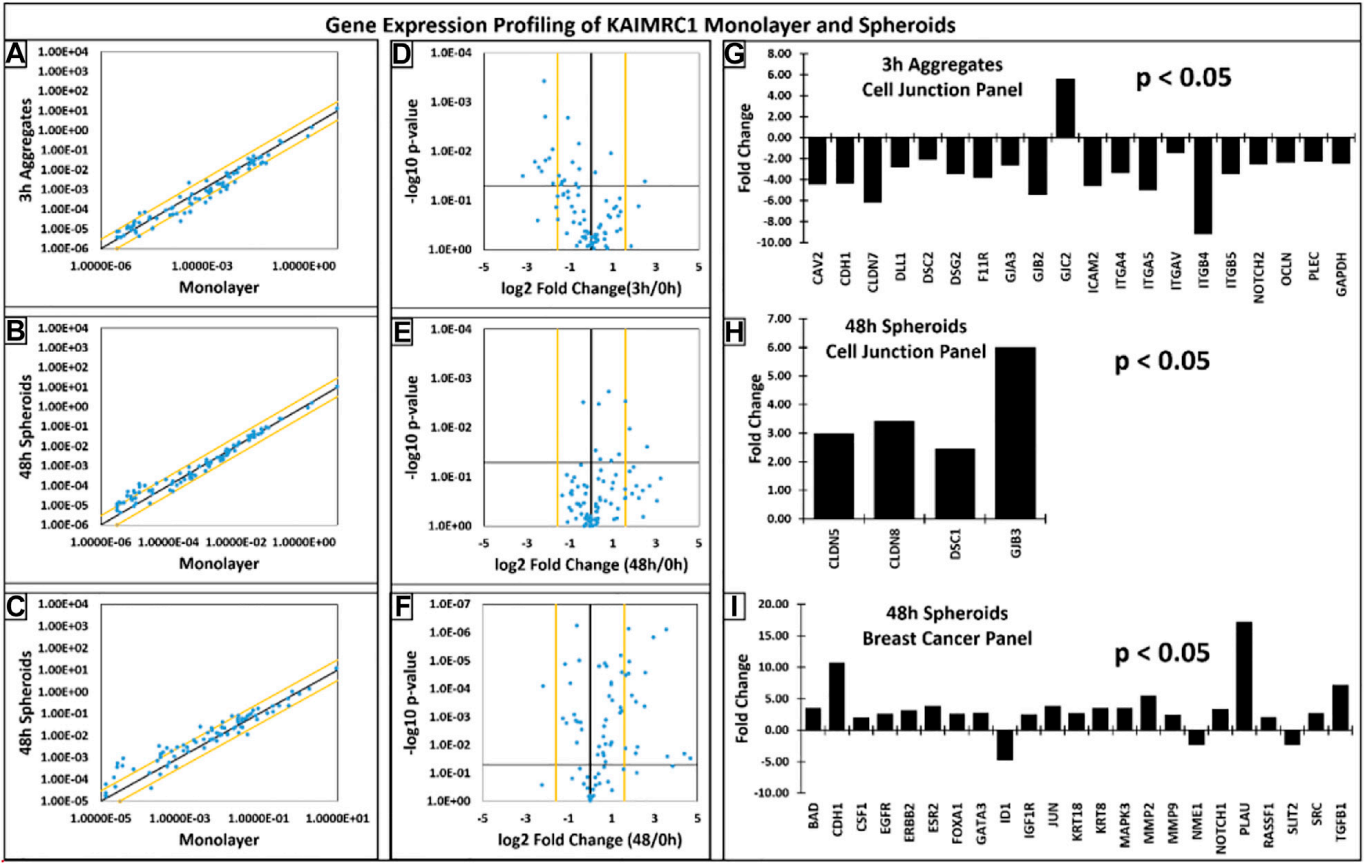
**(A–C)** Scatter plots showcasing the log10 of the expression level of each gene in the spheroids group versus the corresponding value in the monolayer group. The black diagonal line indicates fold changes of 1 or no change. The orange diagonal lines indicate the desired fold-change threshold, i.e., 3. **(D–F)** Volcano plots presenting the log2 of the fold change in each gene’s expression between the groups on the x-axis versus the −log10 of each gene expression changes’ p-value on the y-axis. The vertical black line indicates fold changes of 1 or no change. The orange vertical lines indicate the desired fold-change threshold, which is in 2 in our case. The black horizontal line indicates the desired p-value threshold, i.e., 0.05. **(G–I)** Gene expression profiles of KAIMRC1 spheroids with respect to KAIMRC1 monolayer culture. Qiagen gene array, **(G,H)** cell junction, and **(I)** breast cancer panels were utilized. Several up- and downregulated genes were identified in spheroid culture.

Gene expression profiling of spheroids revealed that most of the key cell adhesion proteins were downregulated during the initial phase of cell aggregation, i.e., 3 h except GJC2. However, high upregulation of key cell junction genes was noticed when spheroids were 48 h old. For instance, CDH1, a gene that makes E-cadherin protein, was significantly downregulated at 3 h, whereas it was highly upregulated at 48 h (Figure 6C). Our immunophenotyping results have also confirmed the upregulation of E-cadherin on protein level.

Interestingly, most of the genes were downregulated during the initial phase of cell aggregation (3 h) except GJC2. High upregulation of key cell junction genes was noticed when spheroids were 48 h old. For instance, CDH1 was significantly downregulated at 3 h, whereas it was highly upregulated at 48 h.

### Applications of KAIMRC1 Spheroids

3D cell culture systems with more than one type of cells mimic more closely the real *in vivo* environment. Here, we have demonstrated the ability of KAIMRC1 spheroids to make 3D co-cultures with other cell types, especially with primary breast cancer-associated fibroblasts (CAFs). We co-cultured KAIMRC1 cells with primary CAFs exhibiting the potential of KAIMRC1 spheroids to grow in co-culture with other cell types (Figure 5B and Supplementary Figure S1). The ultimate goal was to use these co-cultured spheroids in drug discovery applications.

### Drug Response and Sensitivity

**FIGURE 7:**
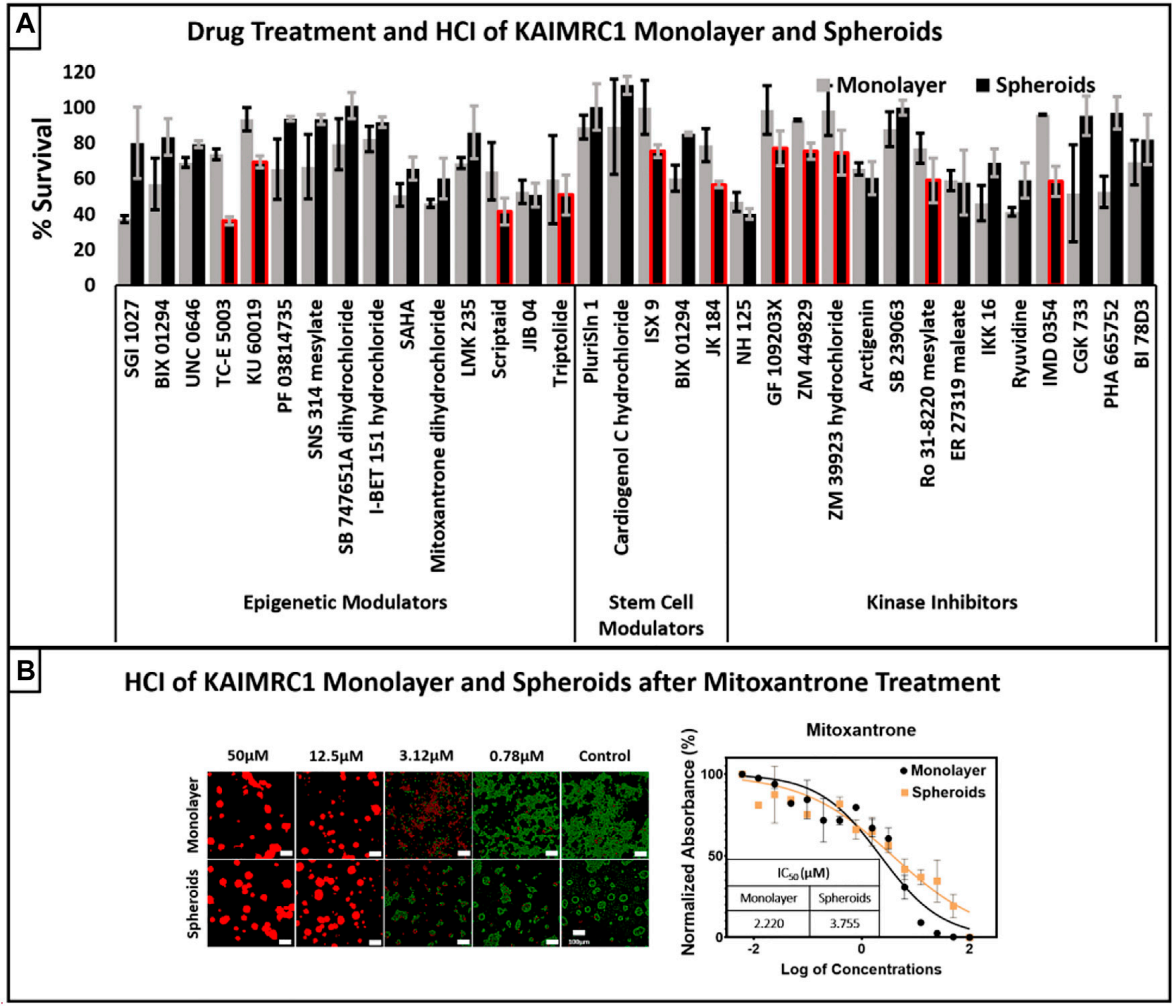
**(A)** Drug treatment and high content imaging of KAIRMC1 monolayer and spheroids. Commercially available toolbox compounds categorized into epigenetics, stem cells, and kinase inhibitors were used to treat KAIMRC1 cells in monolayer and spheroids at 10 µM concentration. The bar graph shows percent survival of cells after treatment for 48 h. It is evident that spheroids showed better survival against most of the compounds. Interestingly, there were several compounds that showed more specificity towards KAIMRC1 spheroids then monolayer (red outlined bars). Error bars denotes ± SD. **(B)** High content imaging of KAIRMC1 monolayer and spheroids after mitoxantrone treatment. KAIMRC1 cells in monolayer and spheroids were treated with graded concentration of mitoxantrone for 48 h. Cells were then stained with blue nuclear dye, green calcein AM live cell marker and propidium iodide (PI) dead cell marker. The graph shows dose–response curve of Mitoxantrone and IC_50_ values for both monolayer and spheroids. *x*-axis = log of mitoxantrone concentration and *y*-axis = normalized absorbance in percentage.

Next, we performed drug treatment and high content imaging of KAIRMC1 monolayer and spheroids. Commercially available toolbox compounds categorized into epigenetics, stem cells, and kinase inhibitors were used to treat KAIMRC1 cells in monolayer and spheroids at 10 µM concentration. The bar graph shows percent survival of cells after treatment for 24 h. It is evident from the results that KAIMRC1 spheroids showed better survival against most of the compounds. Interestingly, several compounds showed more specificity towards KAIMRC1 spheroids than monolayer (Figure 7A). Figure 7B presents high content imaging of KAIRMC1 monolayer and spheroids after Mitoxantrone treatment. KAIMRC1 cells in monolayer and spheroids were treated with a graded concentration of Mitoxantrone for 24 h. High-content image acquisition and analysis software, MetaXpress, was used to analyze monolayer and spheroid images using standard application modules to measure live and dead cells. KIAMRC1 spheroids can be used under environmental control for days with live-cell trackers to monitor cell health kinetics. For instance, we stained KAIMRC1 cells with Live Cell Tracker Green and HOECHST 33342 in monolayer and later changed the serum condition to NBCS to yield spheroids. Post 24 h, cells were treated with drug candidates. The cells in both monolayer and spheroids conformation were imaged for 4 days to monitor the health kinetics.

## Discussion

3D cell culture systems have developed immensely during the last decade, and cell lines that form spheroids need a favorable 3D matrix. There are several techniques available to grow 3D spheroid culture (Achilli et al., 2012; Cui et al., 2017). They are divided into matrix-based natural polymers, such as collagen (Nazari, 2020), hyaluronic acid (Carvalho et al., 2016), chitosan, fibrin (Barsotti et al., 2011) and matrigel (Badea et al., 2019), synthetic polymers, such as polyethylene glycol (PEG) (Han et al., 2015), poly(N-isopropyl acrylamide) (PNIPAM) (Dhamecha et al., 2021), and poly (lactic-co-glycolic acid) (PLGA) (Kuriakose et al., 2019), and matrix-free techniques such as hanging drop (Bartosh and Ylostalo, 2014), bioreactor (Massai et al., 2016), magnetic levitation (Lewis et al., 2017), spinning flasks (Han et al., 2006), and electrical-force-assisted technique (Sebastian et al., 2007). All these techniques require special instrumentation and a lengthy workflow to grow spheroids.

Here, we report a novel method of growing multiple 3D spheroids of KAIMRC1 cells. KAIMRC1 spheroids can be grown in any cell culture treated flask, and 6-, 12-, 24-, and 96-well plates. KAIMRC1 cell monolayers grown in any type of plates can also be used to generate spheroids by changing the FBS- to NBCS-supplemented media. In addition, KAIMRC1 cells can generate cocultures with other cell types such as CAFs (Figure 5B) and immune cells (data not shown). Thus, KIAMRC1 spheroids could be an excellent model to mimic and understand the actual *in vivo* cancer microenvironment.

Cell culture growth mediums are extensively supplemented with serum for decades providing nutrients and growth factors (GFs) for cell growth. GFs are an essential requirement to grow 3D cell cultures. In this work, we have used NBCS instead of FBS, which is rich in GFs, to produce KAIMRC1 spheroids. The NBCS is collected from calves that are <20 days old. It contains macromolecules, attachment and spreading factors, carrier proteins, low molecular weight nutrients, and hormones. The significant difference between FBS and NBCS is the presence of an increased number of antibodies in the NBCS (McGrath et al., 2016; Cheever et al., 2017). The constituents of NBCS are a result of colostrum, the mother’s milk to the calf that is rich in antibodies and GFs such as IGF-I and II (Mero et al., 2002), transforming growth factors α (Okada et al., 1991) β1 and 2 (Tokuyama and Tokuyama, 1993), fibroblast growth factors (Hironaka et al., 1997), epidermal growth factor (Xiao et al., 2002), platelet-derived growth factor, vascular endothelial growth factors (Vuorela et al., 2000), and colony-stimulating factor-1 (Flidel-Rimon and Roth, 1997). Cytokines (Hagiwara et al., 2000), including interleukins, tumor necrosis factor (Rudloff et al., 1992), and chemokines (Maheshwari et al., 2003), are also present in colostrum. Colostrum is also very rich in proteins, vitamin A, and sodium chloride but contains lower carbohydrates, lipids, and potassium than mature milk. The most relevant bioactive components in colostrum are GFs and antimicrobial factors. We hypothesize that KAIMRC1 cells have overexpressed receptors for one or a group of GFs present in the NBCS, which in turn help KAIMRC1 cells to form spheroids during the presence of NBCS in the culture media. We are further analyzing KAIMRC1 cells proteome with and without exposure to NBCS to fish out the overexpressed cell surface receptors. We are also looking into the GFs, cytokines, and hormones that are depleted in the NBCS media after spheroid formation.

Interestingly, phosphorylation of both the insulin receptor (IR) and the IGF-1 receptor (IGF1R) is significantly downregulated in KAIMRC1 spheroids (Figure 4). The binding of both the insulin and insulin-like growth factors IGF-1 to their receptors is known to support cell growth and survival through PI3K and MAPK pathways and metastasis, whereas unliganded IR and IGF1R promote apoptosis (Cao and Yee, 2021). These receptors are known to be overexpressed in several cancer types and reported to be promoting cell survival and proliferation (Lero and Shaw, 2021). Therefore, these receptors are being exploited as anticancer targets, and several clinical trials are underway (Hua et al., 2020). Downregulation of phosphorylation of both these important receptors in KAIMRC1 spheroids is puzzling and needs further research to validate and understand the underlying effect on tumor growth and proliferation. Moreover, our results suggest that phosphorylation of GSK3α/β, ERK, CREB, and Snail is also downregulated, whereas phosphorylation of p53, cJUN, and ChK-2 is significantly upregulated in KAIMRC1 spheroids. ChK-2 and p53 are tumor suppressors and are involved in DNA repair. The phosphorylation of JNK, cJUN, and FAK along with the upregulation of E-cadherin suggests the involvement of JNK/JUN pathway in KAIMRC1 spheroid formation.

It usually takes 2–5 days, depending on the cell type, to produce spheroids using the available techniques (Bresciani et al., 2019). The most promising aspect of KAIMRC1 cells is their ability to grow in both monolayer and spheroids on any treated or non-treated cell culture plastic/glassware within 24 h. This remarkable ability gives KAIMRC1 cells an added advantage over other cell lines, as there is no need for expensive and cumbersome procedures to grow spheroids. The size and amount of the spheroids can also be controlled by adjusting the initial seeding density in a monolayer.

KAIMRC1 spheroids can be used for drug screening and discovery purposes in a high throughput manner. Spheroids simulate penetration barriers that are common in poorly vascularized tumors. Previously, primary sensitivity of spheroids to drugs and resistance associated with repeated exposure have been reported (Sutherland et al., 1981; Langhans, 2018). In this work, we have demonstrated KAIMRC1 spheroids potential as a drug screening tool using HCI. The comparison of KAIMRC1 monolayer versus spheroid has revealed several drug candidates that are either active in monolayer or spheroids only. There were few candidate drugs specifically active against spheroids, especially IMD 0354, an inhibitor of IKKβ that blocks nuclear factor kappa B (NF-KB) translocation to the nucleus and decreases expression of adhesion molecules intracellular adhesion molecule 1 (ICAM-1) and P-selectin (Onai et al., 2004). It is also known to induce cell cycle arrest and apoptosis in breast cancer cells (Tanaka et al., 2006). TC-E 5003, a protein arginine methyltransferase 1 (PRMT1) inhibitor, was also very active against KAIMRC1 spheroids. It has previously been used as an antitumor agent (Zhang et al., 2020) and implicated in oxidative stress, inflammation, and NF-KB translocation to the nucleus (Bissinger et al., 2011). Both the compounds are inhibitors of NF-KB translocation, which reduces the expression of ICAM-1 adhesion molecules and consequently disintegrates and inhibits cell growth in spheroids.

Interestingly, our real-time PCR (RT-PCR) results showcased downregulation of key cell adhesion proteins during the initial phase of cell aggregation for spheroid formation. However, upregulation of most of the cell adhesion proteins was evident after 48 h of spheroid formation. According to our data, PLAU is one of the significantly overexpressed genes found in KAIMRC1 spheroids. PLAU is known to play a role in human cancers, and it promotes cell migration (Liu et al., 2016), invasion, and metastasis (Mahmood et al., 2018; Wu et al., 2021). PLAU gene encodes urokinase-type-plasminogen activator (uPA). The expression of this protein increases many folds in tumor cells compared to normal cells. There are different types of growth factors, hormones, and cytokines that can induce expression of the PLAU gene and morphological changes of the cells (Irigoyen et al., 1999).

Dye penetration is always a problem when it comes to spheroids, and it requires optimization of staining protocols such as the size of the spheroids, reagents, and time required for incubation with the stain. In this regard, KAIMRC1 cells can be instrumental to overcome this issue, as they can be stained in monolayer and then switched to spheroids using the NBCS supplemented media. In this way, fluorescence intensity of the stain remains the same from the core to the outer surface of the spheroids.

The cell-based compound screening assays using 3D cell culture systems instead of 2D may improve cancer drugs’ predictive efficacy. 3D co-culture systems are an excellent alternative to animal models (Bédard et al., 2020). Further advancements in 3D co-culture systems can one day replace animal models to test *in vivo* antineoplastic compound efficacy.

Cancer-associated fibroblasts (CAFs) are rich in alpha-smooth muscle actin (α-SMA), which have been shown to participate in cancer progression (Brivio et al., 2017). In a tumor microenvironment, CAFs are known to have a strong tumor modulating effect (Nurmik et al., 2020) and play a key role in drug resistance. In Supplementary Figure S1, we have shown staining of primary breast CAFs and KAIMRC1 cells separately with fluorescent live-cell trackers before co-culture. Later, the media condition was changed to NBCS to grow cells in co-cultured spheroids. Our next goal is to use these co-cultured spheroids to screen drug candidates, which will add another layer of complexity to the system and eventually bring us one step closer to the actual *in vivo* environment.

In summary, 3D spheroids hold great promise as a tool for disease modeling, target identification, drug screening, and lead identification. Scientists are always on a hunt for new and efficient ways to decipher the complexities of the cellular environment, and spheroids are emerging as a robust system that can complement existing cell lines and animal studies. In addition, the ability of KAIMRC1 cells to switch from monolayer to spheroids within 24 h gives them an edge on other cell lines to be used as a valuable tool for drug discovery purposes.

## Data Availability

All data generated or analyzed during this study are included in this published article [and its supplementary information files]. The KAIMRC1 cell line will be available upon request to any researcher worldwide through King Abdullah International Medical Research Center (KAIMRC). Rules and regulations of the institution will be applicable.
