# Supplementary figures and images for: New Born Calf Serum Can Induce Spheroid Formation in Breast Cancer KAIMRC1 Cell Line
Source: Front Mol Biosci. 2021 Dec 24;8:769030. doi: 10.3389/fmolb.2021.769030 (PMC8740237; doi:10.3389/fmolb.2021.769030)

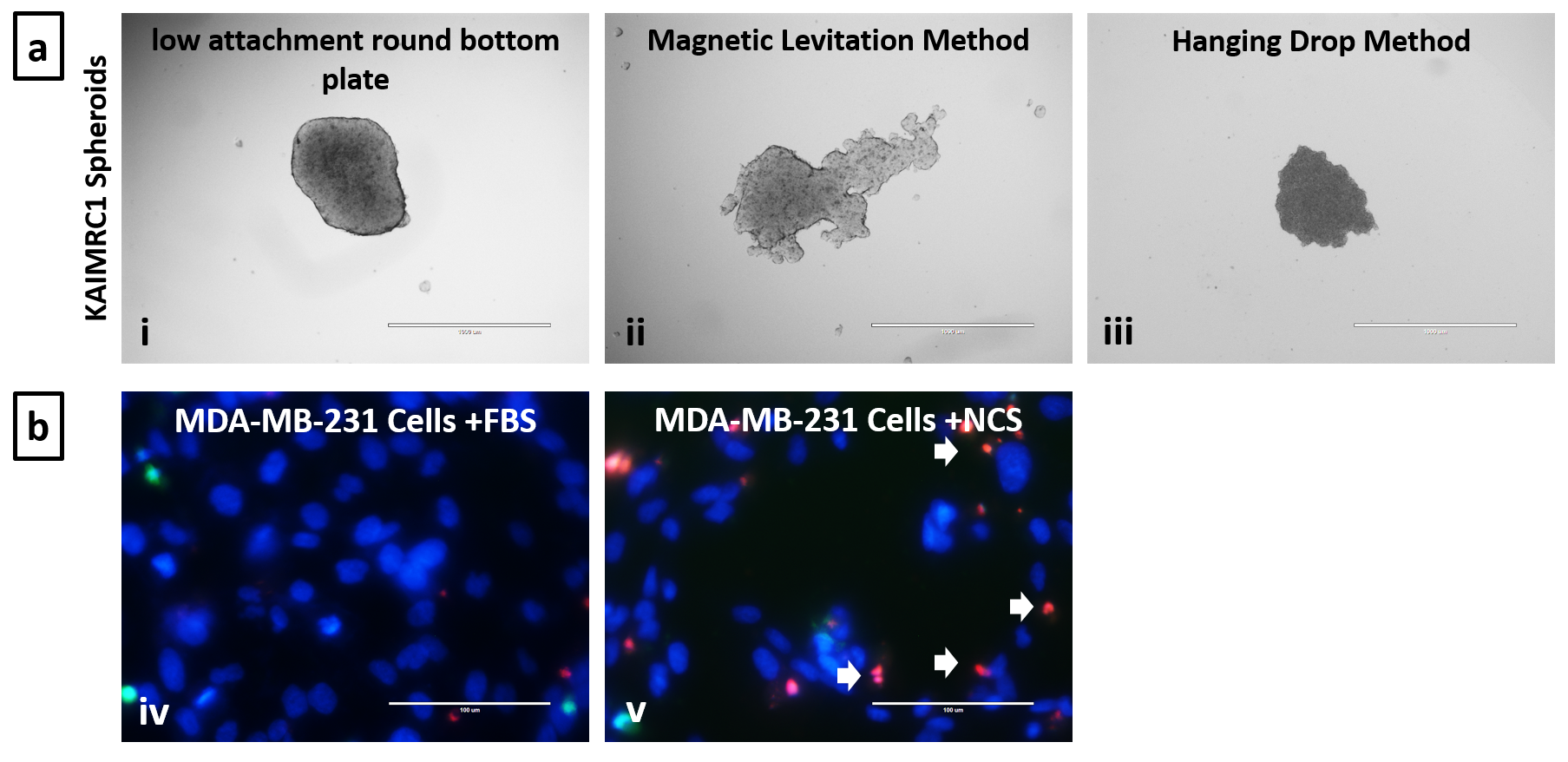

Supplement: Supplementary file 1 [file Image1.tif]
